# Parinaud’s Oculoglandular Syndrome: A Case in an Adult with Flea-Borne Typhus and a Review

**DOI:** 10.3390/tropicalmed5030126

**Published:** 2020-07-29

**Authors:** M. Kevin Dixon, Christopher L. Dayton, Gregory M. Anstead

**Affiliations:** 1 Baylor Scott & White Clinic, 800 Scott & White Drive, College Station, TX 77845, USA; michael.dixon2@bswhealth.org; 2 Division of Critical Care, Department of Medicine, University of Texas Health, San Antonio, 7703 Floyd Curl Drive, San Antonio, TX 78229, USA; dayton@uthscsa.edu; 3 Medical Service, South Texas Veterans Health Care System, San Antonio, TX 78229, USA; 4 Division of Infectious Diseases, Department of Medicine, University of Texas Health, San Antonio, 7703 Floyd Curl Drive, San Antonio, TX 78229, USA

**Keywords:** Parinaud’s, oculoglandular, flea-borne typhus, cat scratch disease, tularemia, lymphadenopathy, granulomatous conjunctivitis

## Abstract

Parinaud’s oculoglandular syndrome (POGS) is defined as unilateral granulomatous conjunctivitis and facial lymphadenopathy. The aims of the current study are to describe a case of POGS with uveitis due to flea-borne typhus (FBT) and to present a diagnostic and therapeutic approach to POGS. The patient, a 38-year old man, presented with persistent unilateral eye pain, fever, rash, preauricular and submandibular lymphadenopathy, and laboratory findings of FBT: hyponatremia, elevated transaminase and lactate dehydrogenase levels, thrombocytopenia, and hypoalbuminemia. His condition rapidly improved after starting doxycycline. Soon after hospitalization, he was diagnosed with uveitis, which responded to topical prednisolone. To derive a diagnostic and empiric therapeutic approach to POGS, we reviewed the cases of POGS from its various causes since 1976 to discern epidemiologic clues and determine successful diagnostic techniques and therapies; we found multiple cases due to cat scratch disease (CSD; due to *Bartonella henselae*) (twelve), tularemia (ten), sporotrichosis (three), *Rickettsia conorii* (three), *R. typhi/felis* (two), and herpes simplex virus (two) and single cases due to tuberculosis, paracoccidioidomycosis, *Yersinia enterocolitica*, *Pasteurella multocida*, *Chlamydia trachomatis*, Epstein–Barr virus, and *Nocardia brasiliensis*. Preauricular lymphadenopathy is a common clinical clue for POGS and is unusual in viral and bacterial conjunctivitis. For POGS, the major etiological consideration is *B. henselae*, which is usually diagnosed by the indirect immunofluorescence serologic technique. Although CSD POGS is usually self-limited, oral azithromycin may hasten resolution. However, other possible etiologies of POGS may also arise from cat or cat flea contact: sporotrichosis, tularemia, *Pasteurella multocida*, or FBT. If there is no cat contact, other epidemiologic and clinical findings should be sought, because several of these conditions, such as tularemia, paracoccidioidomycosis, and tuberculosis, may have grave systemic complications. Although there are usually no long-term ocular sequelae if POGS is properly diagnosed, it still may cause prolonged ocular discomfort and require multiple physician contacts.

## 1. Introduction

Parinaud’s oculoglandular syndrome (POGS) refers to unilateral granulomatous follicular palpebral or bulbar conjunctivitis associated with ipsilateral preauricular and submandibular lymphadenopathy. The lymphadenopathy may extend to the cervical or parotid lymph nodes and may develop concomitantly with the conjunctivitis or its onset may be delayed by several weeks [1]. The physiologic basis of POGS is that the lymphatic drainage of the eyelids leads to the preauricular and submandibular lymph nodes [2]. POGS has been observed in various bacterial, fungal, and viral infections, but most commonly in cat scratch disease (CSD) (due to *Bartonella henselae*), tularemia, and sporotrichosis [3]. Two cases of POGS have been reported in children with flea-borne typhus (FBT) ascribed to *Rickettsia typhi* [4]. (However, it is possible that these cases could have been due to *R. felis* infection because the indirect fluorescent antibody serologic test cannot differentiate between *R. typhi* and *R. felis*) [5]. Herein, we report a case of POGS with mild anterior uveitis in an adult with FBT.

Flea-borne typhus (FBT) commonly presents as a self-limited infection with variable symptoms that may include fever, rash, headache, myalgias, and malaise. The disease causes a systemic inflammatory vasculitis and has been recognized as a cause of a diversity of complications, which include neurologic, renal, pulmonary, and cardiovascular organ dysfunction and failure [6]. Globally, FBT is the most widespread rickettsiosis. It has a predilection for endemicity in port cities, where rats serve as the reservoir. In south Texas and California, FBT remains endemic with reservoirs that include cats, dogs, rodents, and opossums [5].

The aims of the current study are to describe the first case of POGS due to FBT in an adult and to present a diagnostic and therapeutic approach to POGS. In the new millennium, FBT is increasing in incidence in several localities around the globe, so it is important to recognize the diverse manifestations of FBT to make the diagnosis and institute the appropriate treatment. To derive a diagnostic and empiric therapeutic approach to POGS, we reviewed the cases of POGS from its various causes to discern epidemiologic clues and to determine successful diagnostic techniques and therapies.

Methodology. To find cases of Parinaud’s oculoglandular syndrome, PubMed was searched for articles published from 1 January 1976 to 1 May 2020 using the term “oculoglandular.” Additional references were obtained by bibliographic branching and Google Scholar searches with the identical search term. References published in English, German, and Spanish were examined.

## 2. Case

The patient is a 38-year-old Hispanic man with diet-controlled type 2 diabetes, obstructive sleep apnea, depression, and bipolar disorder, who presented to the emergency department with two days of left eye pain and pressure and left-sided submandibular and neck pain. A computerized tomographic scan showed partial opacification of the right ethmoid air cells. Complete blood count and serum chemistry were unremarkable, and throat culture was negative for Group A *Streptococcus*. With presumed sinusitis, he was discharged on amoxicillin/clavulanate for 10 days. Six days later, he presented with continued left facial pain and swelling, left-sided headache, persistent daily fever up to 38.9 °C, rigors, night sweats, myalgias, mattering of the left eye, cough productive of clear sputum, malaise, and an erythematous macular rash on the face, chest, and upper arms. He denied visual changes, sore throat, rhinorrhea, chest pain, abdominal pain, or gastrointestinal symptoms. The patient denied any new sexual contacts, genital ulcers, or dysuria, but admitted to contact with animals (two dogs with fleas, two turtles, and two guinea pigs in the household). He denied opossum exposure (a common reservoir for flea-borne typhus in Texas). The patient reported eating unpasteurized dairy products on a regular basis, as well as having multiple recent mosquito bites. Heart rate on presentation was 124 beats/min, with blood pressure 133/75 mm Hg. On exam, he was found to have left submandibular and preauricular swelling. On admission, the patient was noted to have mild electrolyte abnormalities (hyponatremia, hypokalemia, and hypomagnesemia), mild transaminase elevation, hypophosphatemia, mild proteinuria, and an elevated C-reactive protein. Over the next six days, the patient developed thrombocytopenia, leukocytosis with neutrophilia, and elevated levels of ferritin, procalcitonin, d-dimer, creatinine kinase, and lactate dehydrogenase (Table 1). The international normalized ratio (INR) and serum creatinine were within the normal ranges.

The patient was initially placed on cefepime, metronidazole, and vancomycin by the hospitalist team for sepsis syndrome. Monospot, Epstein-Barr Virus IgM, Cytomegalovirus (CMV) IgM and IgG, human immunodeficiency virus antibody screen and polymerase chain reaction, hepatitis B surface antigen and core IgM, hepatitis A IgM, hepatitis C antibody tests and blood and sputum cultures returned negative. Serologic tests for arboviral encephalitis (California; Eastern and Western Equine), *Bartonella henselae*, *Francisella tularensis*, *Brucella abortus,* and syphilis were negative. Empiric doxycycline was started on hospital day 4, 100 mg by mouth twice daily. After receiving two doses of doxycycline, the patient defervesced, had significant improvement on ophthalmologic exam, and clearance of the rash; he was discharged to finish 14 days of doxycycline. A rickettsial serologic panel was obtained on hospital day 4, and resulted after the patient was discharged; Q fever phase I and II antibodies were negative, *R. rickettsii* IgM and IgG by the indirect fluorescent antibody (IFA) test were both 1:64, and *R. typhi* IgM was >1:256 and the IgG was 1:128 by the IFA test. The ophthalmology service evaluated the patient six days after admission and he was diagnosed with mild anterior uveitis based on grade 1 cellularity in the anterior chamber, trace conjunctival injection, and a decrease in visual acuity in the left eye (20/50 vs. 20/20 in the right eye); he was started on prednisolone drops four times daily. Five days later, the cellularity improved in the anterior chamber of the left eye to rare cells, and the prednisolone was tapered off. At a follow-up appointment 23 days after hospital admission, the patient had completed a 14-day course of doxycycline and reported resolution of all prior symptoms except fatigue, which had significantly improved.

## 3. Discussion

Parinaud’s oculoglandular syndrome (POGS) refers to unilateral granulomatous follicular palpebral or bulbar conjunctivitis associated with ipsilateral preauricular and submandibular lymphadenopathy [1]. Oculoglandular syndrome was first described by French ophthalmologist Henri Parinaud in 1889 when he reported three patients with fever, regional lymphadenopathy, and a follicular conjunctivitis [7]. The early history of the syndrome was discussed in detail by Cassady and Culbertson in 1953 [8]. In our survey of POGS cases since 1976, we found multiple cases due to cat scratch disease (twelve), tularemia (ten), sporotrichosis (three), *Rickettsia conorii* (three), *R. typhi/felis* (two), and herpes simplex virus (two) and single cases due to tuberculosis, paracoccidioidomycosis, *Yersinia enterocolitica*, *Pasteurella multocida*, *Chlamydia trachomatis*, Epstein–Barr virus, and *Nocardia brasiliensis*. Cases of POGS in the older medical literature have been ascribed to syphilis, actinomycosis, blastomycosis, coccidioidomycosis, Chagas disease, mumps, glanders, chancroid, and listeriosis [1,9]. Ophthalmologic involvement in POGS may also include keratitis, vitritis, retinitis, and optic neuritis [1].

### 3.1. POGS Due to Bartonella henselae, the Agent of Cat Scratch Disease

In CSD, the patient typically develops a flu-like illness with local or regional lymphadenopathy. Ocular manifestations occur in 5–10% of CSD cases and POGS is the most common of these [7,10]. Based on four case series of CSD, involving a total of 1589 patients, POGS occurs in 4.3% of cases of CSD [3,10,11,12]. In 1950, Pesme first suggested that POGS was a manifestation of CSD when he observed a child with cat contact that developed conjunctivitis and preauricular lymphadenopathy [13].

**Figure 1:**
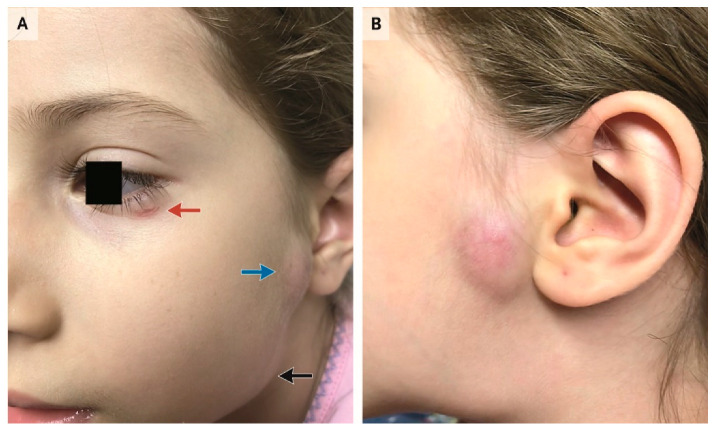
(**A**) Preauricular (blue arrow) and submandibular (black arrow) lymphadenopathy and excoriation below left eye (red arrow) in a 5-year old girl with cat scratch disease Parinaud’s oculoglandular syndrome (CSD POGS); (**B**) lateral view of preauricular and submandibular lymphadenopathy (case #9; [15]).

A cat scratch near the eye or direct conjunctival inoculation with infected flea feces are the most likely inciting events of CSD POGS [7]. (*Bartonella henselae* remains viable in cat flea feces for at least three days [14]). Constitutional symptoms in CSD POGS are usually mild. CSD POGS typically presents as unilateral eye redness, foreign body sensation, and excessive lacrimation [7]. The non-tender conjunctival granuloma of CSD POGS may be initially missed, with the tender preauricular, cervical, or submandibular lymphadenopathy differentiating the condition from a bacterial or viral conjunctivitis [10]. In one series of 24 patients with CSD POGS, preauricular lymphadenopathy was seen in ten patients, submandibular in three, cervical in three, posterior auricular in one, and multiple areas of lymph node involvement in six [13]. Preauricular lymphadenopathy is a common clinical clue for CSD POGS and is unusual in viral and bacterial conjunctivitis (Figure 1). Suppuration of the involved lymph node rarely occurs (two cases out of 48 in one series); typically, the swelling of the involved lymph nodes resolves uneventfully [10].

**Figure 2:**
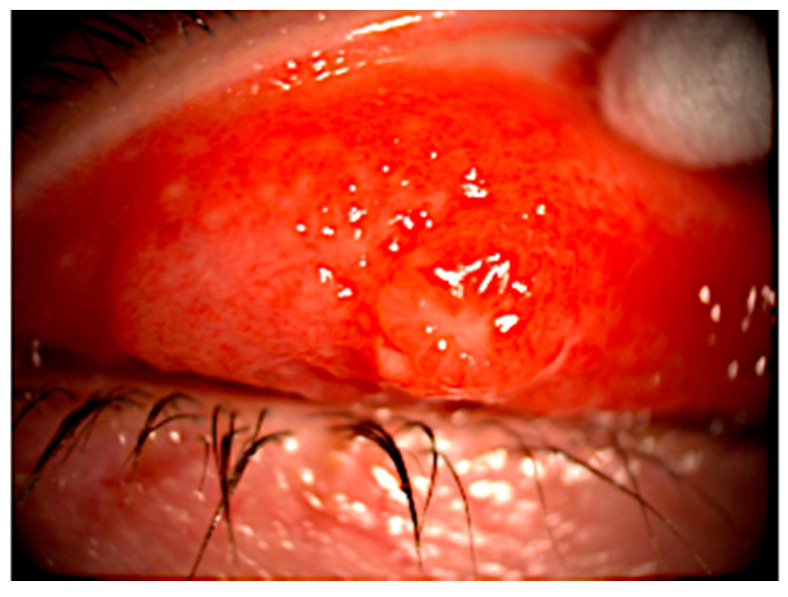
Granuloma in upper tarsal conjunctiva with surrounding papillary reaction in a case of CSD POGS (case #8 [19]).

The ocular discharge tends to be serous; purulent discharge is not observed unless secondary infection has occurred [16]. The conjunctival granulomata usually measure 0.3 to 2 cm in diameter and typically involve the eyelids, but occasionally may be bulbar, forniceal, or medial canthal (Figure 2). Lid swelling may occur, but is usually not painful. Corneal involvement is rare, and is typically limited to superficial punctate keratitis [1]. CSD POGS with concurrent neuroretinitis has also been reported [17,18].

*Bartonella henselae* infection is usually diagnosed by the indirect immunofluorescence serologic technique [19] (*vide infra*). Although CSD POGS is usually self-limited, with the granulomata regressing over a few weeks, antibiotic administration may hasten resolution. Oral azithromycin for 5 days is the regimen of choice (500 mg on day 1, and 250 mg on days 2–5). With concurrent retinal involvement, treatment with doxycycline and rifampin is recommended [20].

The twelve cases of CSD POGS have been described in medical literature since Carither’s landmark 1978 pediatric study [16] are summarized in Table 2. The age of the patients ranged from 5 to 67 years old; 58% were female. Feline exposure was noted in eleven cases. Not all of the cases provided the necessary information to determine an incubation period, but based on cases 2, 3, 7, and 12, it appears to be 1–4 weeks. It was common for patients to suffer with ocular complaints and lymphadenopathy for weeks before presenting for medical care. In 8 of the 12 cases, the diagnosis was made by serologic methods; in two cases, it was based on characteristic histopathologic findings and a history of a cat scratch. In case #3, from 1991, diagnosis was based on the now obsolete Hanger–Rose skin test [21]. In one case, the diagnosis was made by the polymerase chain reaction (PCR) from a conjunctival swab. In two cases (#1 and #2), no antibiotics were given and resolution occurred in two to three weeks; however, in another case, resolution required two months despite presumably effective antibiotics (#6). Case #4, an HIV patient with an unspecified CD4 count, had relapsing infection and required prolonged treatment with two agents. Retinitis (#5), vitritis (#12), and upper lid ptosis (#10) were observed in single cases, but no long-term sequelae were reported in any of the twelve cases.

### 3.2. POGS Due to Francisella tularensis

Tularemia is a zoonosis caused by the bacterium *Francisella tularensis*; it has six clinical syndromes: glandular, ulceroglandular, oculoglandular, oropharyngeal, typhoidal, and pulmonary. Ulceroglandular tularemia is most frequently seen, whereas in most series, the oculoglandular form (OGT; i.e., tularemic POGS) is the least common. The severity of tularemia varies from self-limited to disseminated disease to life-threatening sepsis [29,30]. Tularemia is transmitted by ticks, mosquitoes, biting flies, and contact with various mammals. Rarely, the infection is transmitted by animal bites or by infected water, food, or dusts [29,31].

About 3–5% of cases of tularemia are the oculoglandular form; ocular findings include periorbital edema, conjunctival injection, chemosis, follicular conjunctivitis, and conjunctival epithelial defects [32]. In a series of 105 patients with OGT, other symptoms included fever (87%), sore throat (74%), myalgias (68%), skin ulceration (10%), headache (9%), weight loss (7%), and arthralgias (7%) [33].

*Francisella tularensis* appears as small, Gram-negative coccobacilli on direct stains of exudates; it can be cultured on cysteine-enriched media but growth may be slow (up to ten days or more). Blood cultures are rarely positive. Nevertheless, culture of the organism is a potential hazard to laboratory personnel and therefore other diagnostic methods are preferred. Rapid detection is afforded by direct fluorescent-labelled antibody testing, PCR, and immunochemical antigen detection applied to exudates or biopsy specimens. The microagglutination serologic assay is typically positive ten days after the onset of illness [30].

Streptomycin is the drug of choice for the treatment of tularemia, with gentamicin as an alternative. The aminoglycosides should be administered for 10 days [30]. However, ciprofloxacin for 10 days was found to be more effective than streptomycin in one series [34]. Second-line regimens include tetracyclines or chloramphenicol for 14 days, but treatment failures and relapses occur at a higher rate with these agents as compared to the aminoglycosides. Beta-lactams and macrolides are not recommended. With antibiotic treatment, the overall case fatality rate of tularemia in the United States is less than 2% [30]. Indicators of poor response to treatment are: persistent fever; suppuration and drainage of the affected lymph nodes; an enlargement of the affected lymph nodes; and the appearance of new sites of lymphadenopathy [35]. In one series of 142 cases, 22.5% did not respond to the initial treatment, but there were no deaths [34].

Ten cases of OGT are detailed in Table 3. The cases ranged in age from 13 to 88 years old; 56% were female. Tularemia can be challenging to diagnose because of the plethora of possible exposure routes. Methods of diagnosis were: serologic methods (5/10 cases), PCR (#21), culture (#15), smear of eye exudate (#18), and smear of eye exudate, PCR, and culture in one case (#22). Antibiotic therapy failed in two of the ten cases and lymph node excision was required for resolution (cases #17 and #19). There were no fatalities.

### 3.3. POGS Due to Rickettsial Infections

Although there are many rickettsial species that are pathogenic to humans, POGS has only been observed in *R. typhi/felis* and *R. conorii* infections (Table 4). The former infections are flea-borne, whereas the latter is tick-borne. Two cases of *R. conorii* POGS resulted from careless handling of ticks. The diagnoses were made by serologic methods in all five cases. All of the patients received doxycycline, the drug of choice for rickettsioses, and all cases resolved without sequelae.

### 3.4. POGS Due to Sporotrichosis

Arinella and coworkers described 26 cases of bulbar conjunctival sporotrichosis from Brazil; POGS occurred in 65% of these cases. Twenty-four patients (96%) reported contact with cats with sporotrichosis [47]. Granulomatous conjunctivitis due to *Sporothrix* presents with conjunctival edema, hyperemia, mucopurulent ocular discharge, grouped yellowish conjunctival nodules with a smooth, shiny surface, with or without unilateral lymphadenopathy [48]. In sporotrichosis POGS, preauricular lymphadenopathy was most frequently observed (88%), either alone or with cervical or submandibular involvement. Diagnosis of ocular sporotrichosis is based on direct microscopy and culture. Wet mounts of ocular discharge specimens treated with sodium hydroxide are examined for the presence of cigar-shaped budding yeasts. However, due to the low fungal burden of this infection, the sensitivity of direct visualization is low. For culture, the specimen is plated onto Sabouraud agar with chloramphenicol or Mycobiotic agar, followed by incubation at 25 °C, and observed for 4 weeks for fungal growth. The treatment of choice for sporotrichosis is oral itraconazole at an initial dosage of 100 mg/day for a minimum of 90 days, with discontinuation one month after resolution of ocular disease. The dose was escalated up to 400 mg per day if there was inadequate response to lower doses. All of the clinically evaluable patients in the series of Arinelli and coworkers were cured, but unspecified ocular sequelae occurred in 35% of patients. In hyperendemic areas for sporotrichosis, due to the difficulty of making a definitive diagnosis in a timely manner, empiric treatment has been advocated while awaiting culture results [47].

Three cases of *Sporothrix* POGS were abstracted from the literature (Table 5, cases 28–30). Two of the three patients had cat contact. All patients were diagnosed by culture and were successfully treated with itraconazole, but one case required 60 days of treatment (#30) and another required high-dose itraconazole (#29). No sequelae were noted.

### 3.5. Miscellaneous Causes of POGS

In addition to CSD, tularemia, rickettsial infection, and sporotrichosis, POGS has also been seen in other bacterial infections (*Pasteurella multocida*, *Yersinia enterocolitica*, *Chlamydia trachomatis, Nocardia brasiliensis, Mycobacterium tuberculosis*), viral infections (Epstein-Barr Virus (EBV), Herpes Simplex Virus (HSV)-1), and paracoccidioidomycosis (Table 6). The course of these infections varies widely. In case #31, due to *P. multocida*, the infection persisted for 4 months before an appropriate diagnosis was made and the proper treatment was implemented [52]. The cases due to *Y. enterocolitica* and *C. trachomatis* (#32 and #33, respectively) were complicated by corneal perforation [53,54]. By contrast, in the cases of POGS due to *Nocardia* and *Paracoccidioides* (cases #34 and #39, respectively) [55,56], the infections were quickly diagnosed and treated and resolved uneventfully.

### 3.6. Lymphatic and Ocular Involvement in Flea-Borne Typhus

Both lymphadenopathy and conjunctivitis are relatively uncommon in FBT, occurring in only 13% and 18% of cases, respectively [6]. Other ocular manifestations of FBT are rarely appreciated. Isolated cases of retinitis [61,62], optic neuritis [63,64], hemorrhagic conjunctivitis [65], and uveitis [66] have been reported in FBT. However, in a prospective study of nine consecutive FBT patients who received a comprehensive ophthalmologic evaluation, three patients had ocular signs and symptoms. One patient had ocular redness and slightly blurred vision, one had floaters and slight blurring, and one had a sudden decrease in visual acuity two weeks after fever onset. Furthermore, another five patients had findings on thorough eye exam. Mild vitritis was seen in five of nine patients. Six of the nine patients had an abnormal fundus exam; two patients that had a normal fundoscopic exam had abnormal angiographic results. The posterior chamber findings included white retinal lesions, retinal hemorrhages, retinal vascular leakage, hypofluorescent choroidal dots, optic disc swelling, optic disc staining, and optic neuritis [67]. Howard and Fergie observed two cases of POGS out of 213 pediatric cases of FBT over an 18-year period [68], but POGS has not been previously reported in an adult patient with FBT.

### 3.7. Diagnostic Approach to POGS

Even in the absence of obvious bulbar conjunctivitis, if a patient presents with an unexplained unilateral pre-auricular mass, the upper lid should be everted and examined for follicular conjunctivitis [69]. In a patient with conjunctivitis and ipsilateral preauricular lymphadenopathy, the major etiological consideration is *B. henselae.* Thus, an inquiry about contact with cats should be made. However, other possible etiologies of POGS may also arise from cat or cat flea contact: sporotrichosis [47], tularemia [70], *Pasteurella multocida* [52], or flea-borne typhus [4]. If there is no cat contact, other epidemiologic clues should be sought. Contact with rabbits or ticks would raise concern for tularemia. Tick contact has also been implicated in POGS due to *R. conorii* [44,45,46]. Soil exposure may be a clue to sporotrichosis, nocardiosis, coccidioidomycosis, or paracoccidioidomycosis. Flea-borne typhus should be considered in persons with POGS with possible flea contact or exposure to cats, dogs, rodents, or opossums in an endemic area. In patients with sexual contact, syphilis, herpes simplex, chancroid, and lymphogranuloma venereum have all been implicated. Bilateral conjunctivitis with bilateral preauricular lymphadenopathy is often due to adenovirus [44]. If there is no contact with cats and other epidemiologic circumstances suggest another potential diagnosis, it is important to perform an appropriate diagnostic evaluation because several of these conditions, such as tularemia, coccidioidomycosis, paracoccidioidomycosis, and tuberculosis may have grave systemic complications.

If cat contact is confirmed in a case of POGS, empiric therapy with oral azithromycin for 5 days should be considered. The diagnosis of *B. henselae* infection is usually confirmed by serologic testing by the indirect immunofluorescence assay (IFA), which has a sensitivity and specificity of 90% in immunocompetent persons. In acute *B. henselae* infection, IgM is elevated, but short-lived. For IgG, titers 1:64 or less do not indicate active infection, titers between 1:64 and 1:256 suggest possible infection, and titers >1:256 suggest acute infection [71]. Testing for *B. henselae* infection can also be done by the polymerase chain reaction (PCR); commercial laboratories in the USA conducting this test on blood and tissue in 2020 are ARUP (Salt Lake City, UT, USA) and Quest Diagnostics (Secaucus, NJ, USA). CSD can also be diagnosed by lymph node biopsy with histopathologic exam and Warthin–Starry staining. However, the diagnosis of CSD by culture is impractical because of the slow growth of the organism [71].

Because of the diversity of etiologies of POGS and the difficulty of culturing many of the causative organism, universal (16S rRNA) PCR has been advocated and used successfully to determine the etiology of POGS [42]. The 16S rRNA test is commercially available through the University of Washington Laboratories (Seattle, WA, USA) using frozen tissue specimens, paraffin-embedded tissues, or body fluids. Although there are usually no long-term ocular sequelae if POGS is properly diagnosed, it still may cause weeks of ocular discomfort and necessitate multiple physician contacts.

Various criteria are used to define a confirmed or probable case of FBT. Confirmed cases were defined as a clinically compatible illness with one of the following serologic findings: ≥4-fold rise in antibody titer by IFA between acute and convalescent specimens; or a single IgM or IgG titer of ≥1024 in the endemic area. Probable cases were defined as a clinically compatible illness and a single serologic titer of ≥128 by IFA [72]. The *R. typhi* titers obtained for this patient were IgM >1:256 and IgG was 1:128. The illness was clinically compatible because of presentation with fever, headache, rash, and myalgias; elevated transaminases and lactate dehydrogenase levels; hyponatremia; hypoalbuminemia; thrombocytopenia; lack of responsiveness to beta-lactam treatment; and rapid response to doxycycline. Therefore, this would be defined as a probable case.

## 4. Conclusions

Parinaud’s oculoglandular syndrome should be suspected in patients presenting with unilateral conjunctivitis that is accompanied by regional lymphadenopathy. POGS is most commonly associated with *B. henselae*, but many other organisms have been implicated. POGS associated with FBT has been described in children, but this is the first case described in the literature in an adult patient, which was also accompanied by anterior uveitis. The patient described above had complete symptomatic resolution after 14 days of doxycycline. Transmission likely occurred in this case via manual inoculation after petting a household dog contaminated with feces from infected cat fleas. The diagnosis of FBT in our patient was made on the basis of compatible laboratory and clinical findings, probable flea exposure, positive immunofluorescence serologic tests, and negative laboratory studies for other etiological agents. Thus, *R. typhi*/*R. felis* should be considered in the differential diagnosis for POGS in endemic areas.

## Figures and Tables

**Table 1:** Abnormal laboratory findings of the case patient.

Analyte	Day of Hospitalization	Value	Reference Range	Units
Sodium	1	133	136–145	mmol/L
Potassium	1	3.2	3.5–5.1	mmol/L
Aspartate Aminotransferase	1	71	13–39	IU/L
Alanine Aminotransferase	1	86	7–52	IU/L
C-Reactive Protein	1	14.5	0–1	mg/dL
Magnesium	1	1.8	1.9–2.7	mg/dL
Phosphorous	1	1.5	2.5–5.0	mg/dL
Urine Protein	1	100	0	mg/dL
Platelet Count	3	109	150–400	10^3^/µL
Procalcitonin	3	1.62	<0.5	ng/mL
Creatinine Kinase	3	277	30–233	U/L
Ferritin	5	>1500	10–322	ng/mL
D-Dimer	5	623	0–230	ng/mL
White Blood Cell Count	5	14.5	4–10	10^3^/µL
Lactate Dehydrogenase	6	462	140–271	IU/L

**Table 2:** Cases of cat scratch disease with POGS.

No., [ref], Year	Age (y), Sex	Brief Course
1, [22], 1980	13, F	Cat exposure. Presented with fever, malaise for 2 w. L pa and bilat sm swelling. Palpebral conjunctiva with large granuloma. R submandibular biopsy showed microabscesses and giant cells consistent with CSD. Resolved after 2 w w/o treatment.
2, [23], 1990	50, M	Cat scratch of eyelid, followed in 2 w by unilat eyelid swelling, ipsilat parotid swelling/pa and cervical LAD. Lymph node biopsy showed epithelioid granulomata with foreign body and Langerhans giant cells, with central necrosis. Three weeks after presentation, signs resolved.
3, [21], 1991	49, M	Cat bite on upper lip 4 w prior to presenting with 3-d history of swollen L eyelids, a red eye w/mucopurulent discharge, and tender L pa LAD. Bulbar and tarsal conjunctivae injected w/follicles on the lower palpebral conjunctiva and diffuse papillary reaction. A 3-mm nodule present on bulbar conjunctiva. Tetracycline and topical sulfacetamide prescribed. Conjunctival biopsy interpreted as lymphoma. Warthin–Starry stain negative for *Bartonella.* When the patient returned 3 w later for a second biopsy, the conjunctivitis and LAD had resolved. A Hanger–Rose skin test + for CSD.
4, [24], 1994	29, M	Patient with HIV infection with kitten exposure developed redness and irritation of L eye, tender L pa node and ulcerated nodule of inferior fornix. Received oral cipro for 2 w and topical cipro 4-times a day. Eye redness initially improved but worsened 2 months later. The conjunctival nodule was again present. Failed oral erythromycin and topical tobramycin/dexamethasone. Given 1 month of oral cipro and rifampin with marked improvement. Conjunctiva swab PCR + for *Bartonella*.
5, [18], 1999	38, F	Cat owner; 2-w history of pa LAD; presented with L red eye; L eyelid with subtarsal papillae/large follicles; L bulbar conjunctiva w/granuloma/ulceration, episcleritis; given tobramycin/chloramphenicol eye drops, amox–clav, doxy; + *B. henselae* IgG; mild retinitis; LAD improved in 3 d; all signs and symptoms resolved over 3 w; late growing + culture.
6, [25], 2003	14, F	Contact with kitten. Presented with 12-d history of redness in R eye and pa swelling; received: cephalexin 5 d; amox 4 d; gentamicin 3 d. On exam, conjunctival hyperemia and granuloma on the bulbar conjunctiva; pa and sm LAD. Received spiramycin for 3 d, then clarithromycin for 15 d, and trimethoprim/sulfamethoxazole for 10 d; + *B. henselae* IgG. All symptoms resolved after 2 months.
7, [26], 2004	65, M	Presented with L eye irritation, conjunctival chemosis, and pa LAD. Scratched by kitten one week prior. Granulomatous nodule on palpebral conjunctiva. Initially *B. henselae* seronegative. Given cipro for 4 w; ocular findings improved after 4 days. Three weeks after first negative serologic test, + *B. henselae* IgG.
8, [19], 2017	33, F	History of cat exposure; progressive swelling of L eyelid over 4 w; fever; L pa, sm, parotid, cervical swelling; granulomatous lesion of tarsal conjunctiva; started amox–clav/doxy; + *B. henselae* IgG; stopped amox–clav; finished 2 w of doxy with clinical resolution.
9, [15], 2018	5, F	Cat contact; presented with 2-month history of excoriation below L eye; ipsilat pa, sm LAD; pus aspirated from pa mass; + *B. henselae* IgM; received 5-d azithro; resolved within 2 months.
10, [27], 2018	28, M	Kitten exposure. Presented with headache, progressed to fever, fatigue, myalgia, and conjunctivitis and upper eyelid weakness w/ipsilat pa LAD; + *B. henselae* IgG. Improvement with 5-d azithro.
11, [20], 2019	67, F	Two week history of unilat red eye, chemosis; slit lamp showed a conjunctival granuloma. Topical gatifloxacin/prednisolone acetate initiated; + *B. henselae* IgG. After 1 w, fever and ipsilat pa/sm LAD. Azithro resulted in regression of systemic and ocular disease.
12, [28], 2020	66, F	History of cat scratch of hand, followed 1 w later with R parotid mass/cervical LAD and ipsilat red eye. CT revealed necrotic cervical LAD, necrotic nodules in R parotid gland. Pt had R follicular conjunctivitis and uveitis. Slit lamp showed mild anterior vitritis w/o anterior segment reaction. Parotid biopsy revealed necrotizing granulomas with central stellate microabscesses. *B. henselae* seropositive. Doxycycline given for 10 d. Two months after treatment, there was resolution.
Abbreviations: amox–clav, amoxicillin–clavulanate; azithro, azithromycin; bilat, bilateral; CT, computerized tomograph; cipro, ciprofloxacin; doxy, doxycycline; ipsilat, ipsilateral; pa, preauricular; L, left; LAD, lymphadenopathy; PCR, polymerase chain reaction; R, right; sm, submandibular; unilat, unilateral.		

**Table 3:** Cases of oculoglandular tularemia.

No., [ref], y	Age (y), Sex	Brief Course
13, [36], 1976	67, F	Blood from tick squirted into patient’s eye, causing L conjunctivitis. Failed neomycin/polymyxin B/bacitracin eye ointment x 7-d and 1-d sulfacetamide; then L pa, anterior cervical LAD and corneal clouding. Started intravenous cephalothin and streptomycin and gentamicin eye drops. Initial serology tests negative: *Salmonella*, Weil–Felix, *Brucella, F. tularensis*. Repeat serologic testing positive for *F. tularensis*. Gradual improvement on cephalothin and streptomycin.
14, [37], 2001	17, F	Presented with chills, fatigue, cough, vomiting, diarrhea. One week later, painful nodule on L neck, followed by L eye pain and redness and nodules on legs and arms. Received amox–clav. Developed R conjunctivitis and R-sided cervical LAD. *F. tularensis* seropositive; received 5-d intramuscular streptomycin but fever returned; given methylprednisolone, potassium iodide; eye and skin findings regressed quickly. LAD resolved over 4 months.
15, [38], 2001	18, M	History of rabbit exposure. presented with 4-day history of swollen L upper lid, injected L eye, tender pa, postauricular, and sm LAD, fever, chills, sweats, and headaches. Symptoms persisted despite cephalexin; large granulomatous follicles on L palpebral conjunctivae. A conjunctival culture showed grew *F. tularensis*. Given intramuscular streptomycin and intravenous nafcillin. Over several days, patient’s symptoms and signs improved. Back to baseline health within 2 months.
16, [39], 2012	18, F	L conjunctivitis, periocular ecchymosis, tender pa/sm LAD; *F. tularensis* seropositive; resolved after 21 d of doxy.
17, [40], 2012	44, M	Presented w/headache and swelling of R cheek and eyelids; treated with amox–clav and topical tobra. On exam, follicular conjunctivitis, ulcerating lesion on lower eyelid, and pa LAD. *F. tularensis* detected in aspirate of parotid lymph node. No improvement despite clarithromycin, clindamycin, gent, doxy, cipro for at least one week. The cervical node swelling did not regress and was excised. The ocular symptoms resolved.
18, [40], 2012	68, M	Farmer w/cough and L-sided otalgia; treated with amox–clav; developed fever, L pa/parotid LAD, blepharitis, conjunctival injection, and ocular motility pain. A smear of the exudate indicated *F. tularensis*; recovered after 14 d of doxy.
19, [35], 2013	15, M	One month history of R eye hyperemia, eyelash crusting; developed R pa/sm swelling. + *F. tularensis* seropostiv; placed on a 14-d course of gent. Hyperemia resolved; persistent pa/sm swelling. Placed on streptomycin and cipro. Allergy to cipro, changed to tetracycline. MRI showed involvement of R parotid gland and R masseter. LAD did not resolve despite antibiotics for 62 d, so affected lymph nodes excised, showing necrotizing granulomatous adenitis. Streptomycin stopped 10 day after surgery; resolved after tetracycline for 8 w.
20, [41], 2013	27, F	Pregnant female presented with fever, headache, and generalized pain of a 2-w duration; R purulent conjunctivitis, dacryocystitis, pa/sm LAD; treated with amox–clav, gent eye drops; conjunctivitis resolved in 1 w; dacryocystitis drained; *F. tularensis* seropositive; pt declined systemic antibiotics; dacryocystitis relapsed; drained again; cipro-impregnated sponge placed in surgical site; finished 14-d amox–clav; recovered.
21, [42], 2018	88, M	Presented with R eye pain and discharge; treated with lubricant and anti-inflammatory drops. Two months later, developed painful cervical mass and weight loss. *F. tularensis* detected by PCR of lymph node aspirate. Resolved after 14-d streptomycin.
22, [43], 2019	13, F	Presented with 1 d of fever, sore throat, right papillary conjunctivitis with discharge, swelling of eyelids, sm LAD; started amox–clav; topical ofloxacin and polymyxin/neomycin started. Conjunctival swab, culture, and PCR + for *F. tularensis*. Switched to cipro for 14 d with resolution. Four days before presentation, she was picking goldenrod and rubbed her right eye.
Abbreviations: amox–clav, amoxicillin–clavulanate; gent, gentamicin; ipsilat, ipsilateral; LAD, lymphadenopathy; MRI, magnetic resonance imaging; pa, preauricular; PCR, polymerase chain reaction; sm, submandibular; unilat, unilateral.		

**Table 4:** Parinaud’s oculoglandular syndrome in rickettsial infections.

No., ref, Year	Age (y), Sex	Etiology	Brief Course
23, [4], 2014	11, F	*Rickettsia typhi/felis*	Possible cat exposure. Fever, tender L pa LAD with ipsilat conjunctivitis. Failed clindamycin. L upper lid edema, admitted with vanco/ceftriaxone coverage. Antibiotics changed to azithro on d-2, then discharged on azithro d-3. Returned d-4 with fever, headache, bandemia, elevated AST/ALT. Doxy started. *B. henselae* IgG negative. IgM *R. typhi* 1:64 (convalescent 1:1024). Infection resolved over 10 d.
24, [4], 2014	13, M	*R. typhi/felis*	Admitted with 3 d of fever, R pa swelling with ipsilat conjunctivitis for 6 d; failed clindamycin x 2 d. CT showed 1.5 × 1.5 cm pa LAD with surrounding cellulitis w/o abscess, R facial edema. Bandemia, elevated AST/ALT. Given ceftriaxone. Routine cultures negative. Given gent for possible tularemia on hospital day (HD) 3. Doxy started on HD4. *B. henselae* and *F. tularensis* seronegative. *R typhi* 1:1024 IgG, 1:512 IgM.
25, [44], 1988	15, F	*R. conorii*	Removed ticks from dog, followed in one week by R conjunctivitis and eyelid swelling, pa LAD; then developed fever and rash. Resolved after 7 days of doxy. Serologic testing positive for *R. conorii* infection.
26, [45], 1997	33, F	*R. conorii*	Removing ticks from a dog, splashed blood in her L eye; 1 w later developed L eyelid erythema/swelling, mucopurulent discharge, conjunctival hyperemia, chemosis, a granulomatous nodule on bulbar conjunctiva, small corneal ulcer, and ipsilateral pa/sm LAD. Conjunctival swabs for bacterial, fungal, and chlamydial cultures negative. Started topical chloramphenicol and rolitetracycline and IM piperacillin; developed fever, headache, arthralgia, myalgia, and rash; positive Weil–Felix test. Stopped piperacillin, received doxy for 2 w with complete resolution.
27, [46], 2015	66, M	*R. conorii*	One month history of conjunctivitis L eye, treated with topical tobra, oflox, fusidic acid w/o improvement. On exam, swollen eyelids, conjunctival hyperemia, chemosis, mucopurulent discharge, and ipsilateral pa LAD. Prior to symptoms, eye exposed to contaminated water. Did not recall tick bite. Culture and serology for CSD, syphilis, Lyme Disease, and *Chlamydia* negative. Serologic testing positive for *R. conorii* infection. Resolved after 2-w doxy.
Abbreviations: ac, anterior cervical; amox–clav, amoxicillin–clavulanate; AST/ALT, aspartate aminotransferase/alanine aminotransferase; cipro, ciprofloxacin; doxy, doxycycline; EM, erythema multiforme; LAD, lymphadenopathy; oflox, ofloxacin; pa, preauricular; sm, submandibular; tobra, tobramycin.			

**Table 5:** Parinaud’s oculoglandular syndrome in *Sporothrix* infections.

No., [ref]. Year	Age (y), Sex	Brief Course
28, [49], 2014	21, M	Cat scratch to finger; 2 months later presented with persistent crusted lesion; then developed right eye lesion on tarsal conjunctiva and pa LAD. Fungal culture with *S. schenckii*. Responded to itraconazole.
29, [50], 2002	34, M	Presented w/nodular lesion right eye; received consecutive treatment with topical neomycin–polymyxin B, naphazoline, prednisolone, and tobramycin–dexamethasone over 6 w. Exam showed 1-cm mass on bulbar conjunctiva with episcleral injection and right pa LAD. Biopsy of mass revealed suppurative granulomatous inflammation and yeast. Culture grew *S. schenckii*. Treatment with itraconazole suspension 200 mg each day. Two weeks after biopsy, exam showed multiple conjunctival infiltrates. Started topical fluconazole and itraconazole increased to 300 mg each day; gradual improvement and then resolution.
30, [51], 2016	59, F	History of cat contact. Presented with erythema and pain of left bulbar conjunctiva for 75 days, followed by left cervical and retroauricular LAD and ulcerated nodules of the neck and arms. Culture of eye secretions grew *Sporothrix* sp. Treatment with itraconazole 200 mg/day for 60 days afforded complete resolution.
Abbreviations: LAD, lymphadenopathy; pa, preauricular		

**Table 6:** Parinaud’s oculoglandular syndrome in miscellaneous bacterial, fungal, and viral infections.

No., [ref], Year	Age (y) Sex	Etiology	Brief Course
31, [52], 1987	12, F	*Pasteurella multocida*	History of cat scratch and tick bite 3 w prior. Presented with R keratoconjunctivitis with pa, sm LAD. Failed 4 months of antibiotic therapy. Biopsy with culture of sm lymph node and cornea identified *P. multocida*. Tetracycline, chloramp and corticosteroid eye drops led to almost complete resolution of symptoms.
32, [53], 1977	77, F	*Yersinia enterocolitica*	Presented with R eye swelling/pain; prior drainage of dacryocystic abscess. Found to have diabetic ketoacidosis; developed decreased visual acuity/fistulous tract of medial canthus. R palpebral conjunctiva with follicles/necrotic ulcers. Developed corneal ulcer/pa, sm LAD. Received: IM gentamicin, 14 d; methicillin on days 1/2; carbenicillin on days 2–6; intraocular gent every other day X 6 doses; gent eye drops. Corneal ulcer repaired. Exudate cultures grew *Yersinia enterocolitica*. Ocular inflammation and LAD resolved over 6 w, but corneal graft opacified.
33, [54], 1988	17, F	*Chlamydia trachomatis* L2	Two week history of bilat ocular pain, redness, and discharge; bilat sm, ant cervical LAD; tarsal papillary/follicular reaction; exophytic lesions of bulbar conjunctivae; R eye with corneal perforation. Treated with cefazolin–gent. McCoy cells inoculated w/conjunctival scrapings showed *C. trachomatis* inclusions; + *Chlamydia* IgG; patient had vaginal discharge, inguinal LAD; received 4 w of tetracycline and infection resolved.
34, [55], 1998	56, M	*Nocardia brasiliensis*	Seven days after soil exposure presented with 4-d history of L eyelid swelling; given cefuroxime, 2 d later, developed pa, sm swelling; given IV cefazolin, topical gent; rapid improvement after 5 d of antibiotics; lost to follow-up.
35, [56], 2018	5, M	*M tuberculosis*	Presented with painful R eye, excessive lacrimation, cough, chills, night sweats, weakness, weight loss; painful R pa, sm LAD and bilat inguinal/axillary LAD. HIV test negative; + Mantoux test; eyelid biopsy showed caseating granulomas. Placed on anti-TB therapy; after 2 w, decreased eyelid swelling/conjunctival injection and improved visual acuity.
36, [57], 2008	51, F	EBV	History of rheumatoid arthritis on methotrexate. Three-week history R upper lid edema, conjunctivitis with pa LAD; rash that slowly resolved. Biopsy of tarsal conjunctiva: chronic granulomatous inflammation. Biopsy of lymph node: caseating granuloma; negative bacterial/AFB/fungal stains. Serologic test + for acute EBV infection. Evaluation negative for mumps, CMV, varicella, HSV, parvovirus, ricketttsiae, tularemia, leptospirosis, syphilis, listeriosis, ornithosis, borreliosis, actinomycosis, nocardiosis. Ocular and lymph node manifestations gradually resolved.
37, [58], 2007	14, F	HSV-1	Five day history of L conjunctival injection, periorbital swelling/tenderness, tender ipsilat pa/sm LAD and fever + headache. Vesiculopustules on lower lid developed on hospital day-3. Failed amoxicillin, clarithromycin, topical ofloxacin. Negative bacterial/fungal/*F. tularensis* cultures. *Bartonella* seronegative. Conjunctival swab + HSV-1 by PCR and indirect fluorescent antibody.
38, [59], 1992	17, M	HSV-1	Developed ulcer of R neck 1 w prior to admission. Two days later, had R conjunctival redness and drainage, periorbital swelling and pa, sm LAD. Developed fever, received cephalexin. Pt had R follicular conjunctivitis, and blepharitis with vesiculopustules at lid margin. Cultures from conjunctiva and neck ulcer grew HSV-1. Acyclovir and cefazolin given. Corticosteroid and idoxuridine eye drops given. Defervescence in 48 h. On hospital day 5, conjunctival inflammation markedly reduced and improvement of neck lesion. Oral acyclovir given for 1 more week. Resolution occurred by 3 w after discharge.
39, [60], 2002	31, M	*Paracoc-cidioides braziliense*	Five months of cervical LAD, fever, malaise, weight loss. R eye pain, purulent drainage, R pre- and retroauricular LAD. Lymph node biopsy specimen and sputum grew *P. braziliense*. Improved after 3 w of trimethoprim–sulfamethoxazole.
Abbreviations: ac, anterior cervical; AFB, acid fast bacillus; amox–clav, amoxicillin–clavulanate; cipro, ciprofloxacin; ctx, ceftriaxone; chloramp, chloramphenicol; doxy, doxycycline; EBV, Epstein–Barr virus; IM, intramuscular; L, left; LAD, lymphadenopathy; HSV, herpes simplex virus; pa, preauricular; PCR, polymerase chain reaction; R, right; sm, submandibular; tobra, tobramycin.			

## References

[1] Arjmand P., Yan P., Connor M.D.O. (2015). Parinaud oculoglandular syndrome 2015: Review of the literature and update on diagnosis and management. Clin. Exp. Ophthalmol..

[2] Pan W.R., Suami H., Taylor G.I. (2008). Lymphatic drainage of the superficial tissues of the head and neck: Anatomic study and clinical implications. Plast. Reconstr. Surg..

[3] Ridder G.J., Boedeker C.C., Technau-Ihling K., Sander A. (2005). Cat-scratch disease: Otolaryngologic manifestations and management. Otolaryngol. Head Neck Surg..

[4] Shukla K., Fergie J. (2014). Murine typhus associated with Parinaud’s oculoglandular syndrome in 2 children. Pediatr. Infect. Dis. J..

[5] Anstead G.M. (2020). History, rats, fleas, and opossums. II. The decline and resurgence of flea-borne typhus in the United States, 1945–2019. Interdiscip. Perspect. Infect. Dis..

[6] Tsioutis C., Zafeiri M., Avramopoulos A., Prousali E., Miligkos M., Karageorgos S.A. (2017). Clinical and laboratory characteristics, epidemiology, and outcomes of murine typhus: A systematic review. Acta Trop..

[7] Cunningham E.T., Koehler J.E. (2000). Ocular bartonellosis. Am. J. Ophthalmol..

[8] Cassady J.V., Culbertson C.S. (1953). Cat-scratch disease and Parinaud’s oculoglandular syndrome. AMA Arch. Ophthalmol..

[9] Huang M.C., Dreyer E. (1996). Parinaud’s oculoglandular conjunctivitis and cat-scratch disease. Int. Ophthalmol. Clin..

[10] Carithers H.A. (1985). Cat-scratch disease. An overview based on a study of 1,200 patients. Am. J. Dis. Child..

[11] Daniels W.B., MacMurray F.G. (1954). Cat scratch disease; report of one hundred sixty cases. JAMA.

[12] Murakami K., Tsuk Sasaki K. (2008). Cat scratch disease, an analysis of 130 seropositive cases. J. Infect. Chemother..

[13] Wear D.J., Malaty R.H., Zimmerman L.F., Hadfield T.L., Margileth A.M. (1985). Cat scratch disease bacilli in the conjunctiva of patients with Parinaud’s oculoglandular syndrome. Ophthalmology.

[14] Chomel B.B., Boulouis H.-J., Breitschwerdt E., Kasten R.W., Vayssier-Taussat M., Birtles R.J., Koehler J.E., Dehio C. (2009). Ecological fitness and strategies of adaptation of *Bartonella* species to their hosts and vectors. Vet. Res..

[15] Arango-Ferreira C., Castano J. (2018). Parinaud’s oculoglandular syndrome in cat scratch disease. N. Engl. J. Med..

[16] Carithers H.A. (1978). Oculoglandular disease of Parinaud: A manifestation of cat-scratch disease. Am. J. Dis. Child..

[17] Wong M.T., Dolan M.J., Lattuada C.P., Regnery R.L., Garcia M.L., Mokulis E.C., Labarre R.C., Ascher D.P., Delmar J.A., Kelly J.W. (1995). Neuroretinitis, aseptic meningitis, and lymphadenitis associated with *Bartonella* (*Rochalimaea*) *henselae* infection in immunocompetent patients and patients infected with human immunodeficiency virus type 1. Clin. Infect. Dis..

[18] Grando D., Sullivan L.J., Flexman J.P., Watson M.W., Andrew J.H. (1999). *Bartonella henselae* associated with Parinaud’s oculoglandular syndrome. Clin. Infect. Dis..

[19] Galindo-Bocero J., Sánchez-García S., Álvarez-Coronado M., Rozas-Reyes P. (2017). Parinaud’s oculoglandular syndrome: A case report. Archivos de la Sociedad Española de Oftalmología.

[20] Domínguez I., Cartes C., Sabat P., Ortiz O., Matus G., Traipe L. (2019). Isolated conjunctival granuloma as a first manifestation of Parinaud’s oculoglandular syndrome: A case report. Am. J. Ophthalmol. Case Rep..

[21] Fanous M.M., Margo C.E. (1991). Parinaud’s oculoglandular syndrome simulating lymphoma. Am. J. Ophthalmol..

[22] Loftus M.J., Sweeney G., Goldberg M.H. (1980). Parinaud oculoglandular syndrome and cat-scratch fever. J. Oral Surg..

[23] Jawad A.S., Amen A.A. (1990). Cat-scratch disease presenting as the oculoglandular syndrome of Parinaud: A report of two cases. Postgrad. Med. J..

[24] Le H.H., Palay D.A., Anderson B., Steinberg J.P. (1994). Conjunctival swab to diagnose ocular cat scratch disease. Am. J. Ophthalmol..

[25] Komitova R., Bosheva M., Sander A., Spasova M., Atanasova M. (2003). First case in Bulgaria of Parinaud’s oculoglandular syndrome associated with *Bartonella henselae*. Scand. J. Infect. Dis..

[26] Kymionis G.D., Siganos C.S., Pallikaris I.G. (2004). Late onset of serologic positive titers in a patient with Parinaud’s oculoglandular syndrome. Semin. Ophthalmol..

[27] Valor C., Huber K. (2018). Atypical presentation of cat scratch disease: Parinaud’s syndrome with facial nerve paresis. BMJ Case Rep..

[28] Menezes A.S., Ribeiro D., Lima A.F. (2020). Cat-scratch disease with Parinaud’s oculoglandular syndrome. Turk. Arch. Otorhinolaryngol..

[29] Polat M., Karapinar T., Sirmatel F. (2018). Dermatological aspects of tularaemia: A study of 168 cases. Clin. Exp. Dermatol..

[30] Dennis D.T., Inglesby T.V., Henderson D.A., Bartlett J.G., Ascher M.S., Eitzen E., Fine A.D., Friedlander A.M., Hauer J., Layton M. (2001). Tularemia as a biological weapon: Medical and public health management. JAMA.

[31] Şenel E., Satılmış Ö., Acar B. (2015). Dermatologic manifestations of tularemia: A study of 151 cases in the mid-Anatolian region of Turkey. Int. J. Dermatol..

[32] Gok S.E., Celikbas A.K., Baykam N., Buyukdemirci A.A., Eroglu M.N., Kemer Ö.E., Dokuzoguz B. (2014). Evaluation of tularemia cases focusing on the oculoglandular form. J. Infect. Dev. Ctries..

[33] Erdem H., Yesilyurt M., Karabay O., Elaldi N., Celebi G., Korkmaz N., Güven T., Sümer S., Tulek N., Ural O. (2014). Evaluation of tularaemia courses: A multicentre study from Turkey. Clin. Microbiol. Infect..

[34] Pérez-Castrillón J.L., Bachiller-Luque P., Martín-Luquero M., Mena-Martín F.J., Herreros V. (2001). Tularemia epidemic in northwestern Spain: Clinical description and therapeutic response. Clin. Infect. Dis..

[35] Kosker M., Okur D.S., Kilic O., Akil F., Yilmaz M., Ozturk O., Cokugras H.C., Camcioglu Y., Akcakaya N. (2013). A case of oculoglandular tularemia resistant to medical treatment. Scand. J. Infect. Dis..

[36] Guerrant R.L., Humphries M.K., Butler J.E., Jackson R.S. (1976). Tickborne oculoglandular tularemia: Case report and review of seasonal and vectorial associations in 106 cases. Arch. Intern. Med..

[37] Peter R., Banyai T. (2001). Erythema nodosum revealing oculoglandular tularemia. Dermatology.

[38] Thompson S., Omphroy L., Oetting T. (2001). Parinaud’s oculoglandular syndrome attributable to an encounter with a wild rabbit. Am. J. Ophthalmol..

[39] Altuntas E.E., Polat K., Durmuş K., Uysal I.Ö., Müderris S. (2012). Tularemia and the oculoglandular syndrome of Parinaud. Braz. J. Infect. Dis..

[40] Zamboni S.L., Kipfer-Kauer A., Knect P.B. (2012). Tularemia as a rare cause of Parinaud’s oculoglandular syndrome. Klin. Monatsbl. Augenh..

[41] Celik T., Yuksel D., Kosker M., Turkoglu E.B. (2013). Unilateral acute dacryocystitis associated with oculoglandular tularemia: A case report. Semin. Ophthalmol..

[42] Donate-Pérez-Molino P., Castelló-Abietar C., Fernández-Suárez J., de Vicente J.C. (2018). Tularemia: Diagnosis of an unexpected oculoglandular case in a non-endemic area by universal PCR. Enferm. Infecc. Microbiol. Clin..

[43] Frischknecht M., Meier A., Mani B., Joerg L., Kim O.C.-H., Boggian K., Strahm C. (2019). Tularemia: An experience of 13 cases including a rare myocarditis in a referral center in Eastern Switzerland (Central Europe) and a review of the literature. Infection.

[44] Espejo E., Bella F., Espaulella J., Romanillos T. (1988). Mediterranean spotted fever presenting as oculoglandular syndrome. Trans. R. Soc. Trop. Med. Hyg..

[45] Pinna A., Sotgiu M., Carta F., Zanetti S., Fadda G. (1997). Oculoglandular syndrome in Mediterranean spotted fever acquired through the eye. Br. J. Ophthalmol..

[46] Abroug N., Khairallah-Ksiaa I., Kahloun R., Khochtali S., Zaouali S., Khairallah M. (2015). Parinaud’s oculoglandular syndrome revealing subclinical *Rickettsia conorii* infection. Int. Ophthalmol..

[47] Arinelli A., do Couto Aleixo A.L.Q., Freitas D.F.S., do Valle A.C.F., Almeida-Paes R., Gutierrez-Galhardo M.C., Curi A.L.L. (2019). Ocular sporotrichosis: 26 cases with bulbar involvement in a hyperendemic area of zoonotic transmission. Ocul. Immunol. Inflamm..

[48] Yamagata J.P.M., Rudolph F.B., Nobre M.C.L., Nascimento L.V., Sampaio F., Arinelli A., Freitas D.F. (2017). Ocular sporotrichosis: A frequently misdiagnosed cause of granulomatous conjunctivitis in epidemic areas. Am. J. Ophthalmol. Case Rep..

[49] Ferreira C.P., Nery J.A.D., De Almeida A.C.O., Ferreira L.C., Côrte-Real S., Conceição-Silva F. (2014). Parinaud’s oculoglandular syndrome associated with *Sporothrix schenckii*. IDCases.

[50] Hampton D.E., Adesina A., Chodosh J. (2002). Conjunctival sporotrichosis in the absence of antecedent trauma. Cornea.

[51] Medeiros K.B., Landeiro L.G., Diniz L.M., Falqueto A. (2016). Disseminated cutaneous sporotrichosis associated with ocular lesion in an immunocompetent patient. An. Bras. Dermatol..

[52] Balster L., Bopp S. (1987). Oculoglandular syndrome (Parinaud) caused by *Pasteurella multocida* with corneal involvement. A severe clinical course. Fortschr. Ophthalmol..

[53] Chin G.N., Noble R.C. (1977). Ocular involvement in *Yersinia enterocolitica* infection presenting as Parinaud’s oculoglandular syndrome. Am. J. Ophthalmol..

[54] Buus D.R., Pflugfelder S.C., Schnachter J., Miller D., Forster R.K. (1988). Lymphogranuloma venereum conjunctivitis with a marginal corneal perforation. Ophthalmology.

[55] Gardam M.A., Arthurs B.P., Miller M.A. (1998). An eye for horticulture. Lancet.

[56] Mataswa N., Masanganise R.T. (2018). Tuberculosis manifested as Parinaud’s oculoglandular syndrome. Afr. Vis. Eye Health.

[57] Charbel Issa P., Eis-Hubinger A.M., Klatt K., Holz F.G., Loeffler K.U. (2008). Oculoglandular syndrome associated with reactivated Epstein-Barr-virus infection. Br. J. Ophthalmol..

[58] Parentin F., Molin G.D., D’Agaro P., Busetti M., Campello C. (2007). Parinaud’s oculoglandular syndrome due to herpes simplex virus type 1. Ocul. Immunol. Inflamm..

[59] Caputo G.M., Byck H. (1992). Concomitant oculoglandular and ulceroglandular fever due to herpes simplex virus type I. Am. J. Med..

[60] Costa P.S., Hollanda B.V., Assis R.V., Costa S.M., Valle L.M. (2002). Parinaud’s oculoglandular syndrome associated with paracoccidioidomycosis. Revista do Instituto de Medicina Tropical de São Paulo.

[61] Hudson H.L., Thach A.B., Lopez P.F. (1997). Retinal manifestations of acute murine typhus. Int. Ophthalmol..

[62] Lu T.M., Kuo B.I., Chung Y.M., Liu C.Y. (1997). Murine typhus presenting with multiple white dots in the retina. Scand. J. Infect. Dis..

[63] Zhang J., Pau D., Lee A.G. (2011). Postinfectious optic neuropathy in endemic typhus. J. Neuroophthalmol..

[64] Espino Barros Palau A., Morgan M.L., Lee A.G. (2014). Bilateral optic atrophy in endemic typhus. Can. J. Ophthalmol..

[65] Chueng T.A., Koch K.R., Anstead G.M., Agarwal A.N., Dayton C.L. (2018). Case report: Early doxycycline therapy for potential rickettsiosis in critically ill patients in flea-borne typhus-endemic areas. Am. J. Trop. Med. Hyg..

[66] Beltrán L.M., García S., Vallejo A.J., Bernabeu-Wittel M. (2011). Bilateral anterior uveitis and *Rickettsia typhi* infection. Enferm. Infecc. Microbiol. Clin..

[67] Khairallah M., Ben Yahia S., Toumi A., Jelliti B., Loussaief C., Romdhane F.B., Messaoud R., Chakroun M. (2009). Ocular manifestations associated with murine typhus. Br. J. Ophthalmol..

[68] Howard A., Fergie J. (2018). Murine typhus in South Texas children: An 18-year review. Pediatr. Infect. Dis. J..

[69] Pick W. (1956). Parinuad’s oculoglandular syndrome. J. Pediatr..

[70] Capellan J., Fong I.W. (1993). Tularemia from a cat bite: Case report and review of feline-associated tularemia. Clin. Infect. Dis..

[71] Mabra D., Yeh S., Shantha J.G. (2018). Ocular manifestations of bartonellosis. Curr. Opin. Ophthalmol..

[72] Murray K.O., Evert N., Mayes B., Fonken E., Erickson T., Garcia M.N., Sidwa T. (2017). Typhus group rickettsiosis, Texas, USA, 2003–2013. Emerg. Infect. Dis..

